# The prediction of discharge from in-patient psychiatric rehabilitation: a case-control study

**DOI:** 10.1186/1471-244X-11-149

**Published:** 2011-09-16

**Authors:** Joanna Bredski, Andrew Watson, Debbie A Mountain, Fiona Clunie, Stephen M Lawrie

**Affiliations:** 1 Rehabilitation Service, Royal Edinburgh Hospital, Edinburgh, UK; 2 Intensive Psychiatric Care Unit, Royal Edinburgh Hospital, Edinburgh, UK; 3 Division of Psychiatry, University of Edinburgh, Edinburgh, UK

## Abstract

**Background:**

At any time, about 1% of people with severe and enduring mental illness such as schizophrenia require in-patient psychiatric rehabilitation. In-patient rehabilitation enables individuals with the most challenging difficulties to be discharged to successful and stable community living. However, the length of rehabilitation admission that is required is highly variable and the reasons for this are poorly understood. There are very few case-control studies of predictors of outcome following hospitalisation. None have been carried out for in-patient rehabilitation. We aimed to identify the factors that are associated with achieving discharge from in-patient rehabilitation by carrying out a case-control study.

**Methods:**

We compared two groups: 34 people who were admitted to the Rehabilitation Service at the Royal Edinburgh Hospital and discharged within a six year study period, and 31 people who were admitted in the same period, but not discharged. We compared the groups on demographic, illness, treatment and risk variables that were present at the point of their admission to rehabilitation. We used independent t tests and Pearson Chi-Square tests to compare the two groups.

**Results:**

We found that serious self harm and suicide attempts, treatment with high dose antipsychotics, antipsychotic polypharmacy and previous care in forensic psychiatric services were all significantly associated with non-discharge. The non-discharged group were admitted significantly later in the six year study period and had already spent significantly longer in hospital. People who were admitted to rehabilitation within the first ten years of developing psychosis were more likely to have achieved discharge.

**Conclusions:**

People admitted later in the study period required longer rehabilitation admissions and had higher rates of serious self harm and treatment resistant illness. They were also more likely to have had previous contact with forensic services. This change over time is likely to be due to the drive in Scotland to manage mentally disordered offenders in conditions of lower security. There is a growing need for secure longer-term in-patient rehabilitation, particularly for people previously treated in forensic services. Admission to rehabilitation earlier in a person's illness may improve their outcome.

## Background

At any time, about 1% of people with severe and enduring mental health problems such as schizophrenia require in-patient psychiatric rehabilitation [1]. Most people are referred to rehabilitation because they have not recovered enough to leave hospital, despite receiving treatment as recommended by the National Institute for Health and Clinical Excellence (NICE) treatment algorithm [1,2]. Discharge from in-patient rehabilitation is a measure of good outcome because it marks an important stage in the individual's recovery. The person will have gained the skills they need for daily living and for managing their own illness. Their environment will have been adjusted to minimise disability and handicap. They will be able to engage with community support and will have been supported to regain hope, agency and a sense of identity [3]. With appropriate treatment in rehabilitation even individuals with highly challenging difficulties can move on to successful and stable community living [4,5]. Community living improves quality of life and social functioning and is preferred by patients [4,5]. Hospital beds are expensive and community care is more cost-effective than repeated admission [6]. Discharge from in-patient rehabilitation that results in successful and stable community living is likely to be cost-effective [6].

International studies of schizophrenia and other psychotic disorders have found only limited evidence that demographic, illness and treatment variables predict outcome, either in terms of remission or disability [7-11]. The relevant UK literature on predicting outcome after hospitalisation is mainly composed of cross-sectional studies of long-stay patients in acute general psychiatric wards. The definition of long-stay in the literature varies, but is usually defined as a stay of either over six months or one year. UK studies have found that long-stay is associated with schizophrenia, violence and the need for re-housing [12-15]. However, there are very few case-control studies of long-stay in the literature, and only one from the UK [16]. This study was carried out in London and compared 47 people who were admitted to acute general and intensive psychiatric care wards for over six months with next admission controls. Their strongest finding was that cases were more severely ill than controls. The in-patient rehabilitation population is unlike the acute general population as in rehabilitation every patient has a severe and enduring mental illness. We identified a gap in the literature for a case-control study of outcome following admission to in-patient rehabilitation. We aimed to address this gap by carrying out a case-control study to identify the factors that are associated with achieving discharge from in-patient rehabilitation. Knowledge of the factors that are associated with outcome can be used to guide treatment for individual patients. It can also be used at a service level to optimise the structure of services to meet the needs of the patient population.

## Methods

### Setting

NHS Lothian provides services for 800,000 people in Edinburgh and the Lothians and is the second largest NHS board in Scotland. The Rehabilitation Service at the Royal Edinburgh Hospital consists of an in-patient service with four wards and a total of 74 beds and a Community Rehabilitation Team. Two wards with 25 beds and 15 beds respectively offer high-dependency rehabilitation. This is for people with a high level of symptoms as well as significant risk histories and challenging behaviours. The two other wards provide longer-term complex care. This offers longer term admission, often for several years. This is for people with a high level of disability from a complex mix of conditions who also present a risk to themselves or others. One is a 19 bedded male only ward and the other a 15 bedded ward that provides a service for people with mental illness as well as serious physical health problems. Wolfson, Holloway and Killaspy have written a full description of the types of in-patient rehabilitation elsewhere [17]. There was no change in the ward mix during the study period. The Service accepts referrals for people with all types of mental disorder including, unusually for rehabilitation services, borderline personality disorder. Most referrals are from in-patient wards in the general adult acute service at the Royal Edinburgh Hospital. Another large source of referrals is the Orchard Clinic, which is one of two medium secure forensic units currently in Scotland. A smaller number of people are referred by community mental health teams. West Lothian has 12 in-patient rehabilitation beds in a community rehabilitation unit within a hospital site. East Lothian and Midlothian each have an eight bedded community rehabilitation unit managed by the independent sector, but will refer to the Rehabilitation Service if more intensive rehabilitation is required. In England there has been a rapid rise in the independent sector provision of in-patient residential and nursing care. These are often far from a person's local area and have become known as out-of-area treatments. In England, 21% of residential and nursing care placements are in out-of-area treatments and these cost on average 64% more than local placements [18]. Although out-of-area placements are common and highly topical in England, they are not common in Scotland. During the study period no patients who required in-patient rehabilitation were placed in out-of-area treatments.

### Sample

The sample consisted of two groups. Both of the groups were admitted to the Rehabilitation Service wards at the Royal Edinburgh Hospital in a six year period beginning 1^st ^April 2004 and ending 1^st ^April 2010. The first group were admitted and discharged within the same period (n = 34). Many more people were discharged from the Service during this period, but only those admitted on or after 1^st ^April 2004 were included in the study. The second group were admitted during the same time period, but had not achieved discharge by the end of the six years we studied (n = 31). This group of non-discharged patients was selected on April 1^st ^2010 and matched to the discharged group by ward of admission. There were no exclusion criteria.

### Data sources

In April 2004 a new set of baseline assessments was introduced by the Rehabilitation Service. These 20 page documents record the person's psychiatric, personal and past medical history as well as a risk assessment and information about their medication and physical health. The documents are produced by the multi-disciplinary team within the first two months of admission and are stored electronically. We designed data collection sheets for the study to gather data from these documents. The data gathering sheets are available from the first author on request. The date of discharge was gathered from a computerised patient management system.

### Method

We used a case control study design to compare the two groups described above on demographic, illness, social, treatment and risk variables that were present at the point of their admission to in-patient rehabilitation. We chose these variables based on the literature around long-stay. Only variables that were reliably recorded were chosen.

### Statistical analysis

Independent t tests were used to compare the groups on continuous, normally distributed variables including age, date of admission and length of admission to rehabilitation. Pearson Chi-Square tests were used for categorical data and compared the groups on all other variables. The data was collected and analysed by the lead author. We consulted a statistician before analysing the data and carried out the analysis using Minitab for Windows.

### Ethical approval

The South East Scotland Research Ethics Service confirmed that ethical approval was not required under NHS research governance arrangements.

## Results

### Characteristics of patients

There were no significant differences between the two groups in terms of age, sex or diagnosis. This can be seen in table 1. The non-discharged group were admitted significantly later in the study period (t = 3.8, P = 0.0003) and had already spent a significantly longer time in hospital (t = 2.2, P = 0.03).

**Table 1:** Baseline characteristics on admission and discharge status

	Outcome status	
	
	Discharged	Non-discharged
Variable	(n = 34)	(n = 31)
Age, years: mean (s.d.)	35.8 (12.3)	39.1 (11.7)

Sex, n		
Males	23	22
Females	11	9

Diagnosis^1^, n		
Schizophrenia (any type)	29	26
Schizoaffective disorder	2	3
Bipolar affective disorder	1	1
Other psychotic illness	2	0
Alcohol related brain damage	0	1

Date of admission, mean*	14-Oct-06	03-Jan-08

Length of admission, years: mean* (s.d.)	1.4 (0.8)	2.2 (1.3)

1. Due to small numbers, schizophrenia was compared against all other diagnoses grouped together.

* P < 0.05

### Risk factors

Table 2 shows that a history of self harm or suicide attempts (χ^2 ^= 4.7, P = 0.03) and previous care in forensic psychiatric services (χ^2 ^= 5.7, P = 0.02) were both significantly associated with non-discharge. Aggression, absconding and sexual offences or incidents (for example sexual disinhibition or other inappropriate sexual acts that did not result in prosecution) were also more common in the non-discharged group.

**Table 2:** Risk variables on admission and discharge status

	Outcome status	
	
	Discharged	Non-discharged
Risk	(n = 34)	(n = 31)
Self harm/suicide attempts, n*	17	24
Previous forensic care, n*	2	9
Aggression, n	23	26
Sexual offences/incidents, n	9	14
Disengagement, n	29	25
Absconding, n	14	20
Previous prison stay, n	8	5
Alcohol dependence, n	2	4
Other substance dependence, n	3	5
Harmful use of alcohol, n	14	16
Harmful use of other substances, n	14	16

* P < 0.05

Harmful or dependent substance use was very common and the rates were similar in the two groups. Alcohol dependence was present in 9% and opiate dependence in 11% of the total sample and harmful use of either was present in 46%.

### Treatment factors

Table 3 shows that the prescription of clozapine, either at the point of admission to rehabilitation or ever in the past, was not associated with discharge. However, the prescription of high dose antipsychotic medication in the past was significantly associated with non-discharge (χ^2 ^= 6.6, P = 0.01). Unfortunately in 15 of the discharged group and 11 of the non-discharged group (40% of the whole sample) it was not clear whether or not high dose antipsychotics had been prescribed in the past. These cases were excluded from the comparison. Antipsychotic polypharmacy was defined as the prescription of more than one regular antipsychotic. Polypharmacy in the past was significantly associated with non-discharge (χ^2 ^= 5.7, P = 0.02). There was no association between compulsory treatment under the Mental Health Act (Care & Treatment) (Scotland) Act 2003 and outcome.

**Table 3:** Variables relating to previous treatment on admission and discharge status

	Outcome status	
	
	Discharged	Non-discharged
Treatment variables	(n = 34)	(n = 31)
Clozapine on admission, n	13	15
Clozapine ever, n	20	20
High dose on admission, n	6	6
High dose ever, n*	5	10
Antipsychotic polypharmacy on admission, n	4	9
Antipsychotic polypharmacy ever, n*	13	21
Subject to compulsory treatment, n	22	21

*P < 0.05

### Illness and social factors

There were no statistically significant differences between the groups in the illness and social factors we studied (see table 4). The discharged group were more likely to have been admitted to rehabilitation within ten years of their first presentation with psychosis, but the difference was not statistically significant (χ^2 ^= 2.4, P = 0.12).

**Table 4:** Illness and social variables on admission and discharge status

	Outcome status	
	
	Discharged	Non-discharged
Variable	(n = 34)	(n = 31)
Age at onset psychosis, years: mean (s.d.)	23.0 (7.6)	23.4 (7.9)

Admission during first 10y of psychosis, n	18	13

Diagnosed affective component, n	11	16

Family history		
Psychotic illness, n	16	10
Other mental illness, n	17	10
Substance dependence, n	9	6

Social factors		
Homelessness ever, n	11	8
Paid employment, ever, n	22	22
Supported accommodation, ever, n	12	15
Educational qualifications, any, n	12	20

Early life abuse or neglect^1^, n	9	7

Carer's view present, n	13	12

Accommodation prior to admission^2^		
Parental home, n	11	6
Supported accommodation, n	5	5
Temporary accommodation, n	5	8

1. Early life abuse or neglect was recorded as present in 16 cases, absent in two cases and in all other cases presence or absence was not recorded.

2. This refers to the type of accommodation the person was resident in before admission to hospital rather than before admission to rehabilitation. Only four people were admitted directly to rehabilitation from the community.

Surprisingly small numbers had a recorded history of abuse or neglect in childhood. Only four had a recorded history of childhood sexual abuse. It is likely that abuse and neglect in childhood was more common than this, but that it had not been disclosed or recorded. We looked at whether a carer's view was recorded as a proxy measure for carer involvement in treatment decisions. There was no significant relationship between discharge and whether a carer's view was recorded. Interestingly, more educational qualifications were held by the non-discharged group (P = 0.07). Three of the four university degrees conferred were to this group.

## Discussion

We found that a history of self harm or suicide attempts, treatment with high dose antipsychotics and antipsychotic polypharmacy were all significantly associated with non-discharge. Previous treatment in forensic psychiatric services was also associated with non-discharge. The non-discharged group were admitted significantly later in the six year study period and had already spent significantly longer in hospital.

### Risk factors

A history of self harm or suicide attempts was significantly associated with non-discharge. Aggression and sexual offences or incidents were also more common in the non-discharged group. Self harm and suicide attempts before and after admission to hospital have been shown to increase the risk of suicide in people with schizophrenia [19]. Challenging behaviours, such as self harm and aggression, are reasons for discharge to the community not to be considered [20]. Improvements in challenging behaviours appear to be more important than changes in symptoms in allowing discharge to the community [20]. In a study of 72 long-stay patients who were considered unsuitable for community living there was no improvement in challenging behaviours at the end of the first year of rehabilitation. However, after five years there was a significant reduction in challenging behaviours and this allowed 40% of the patients to be discharged to supported accommodation in the community [20]. A slower pace of rehabilitation may be required to put into place the behavioural programmes that allow challenging behaviours to diminish. We also found that previous admission to forensic psychiatric services was significantly associated with non-discharge. In Edinburgh, most of those transferred from forensic services come from forensic rehabilitation wards in a medium secure unit. They are transferred to psychiatric rehabilitation because their needs cannot be met in the community. This could be due to challenging behaviours, vulnerability or difficulty in gaining the skills that they need for community living. As a group they are likely to require different interventions, often within the Care Programme Approach for restricted patients, as well as a slower pace in rehabilitation.

Harmful or dependent use of substances was more common in the sample than in the general population. In 2006 in Scotland 1.6% of people aged between 15 and 64 had dependent use of opiates or benzodiazepines [21]. In our sample opiate dependence was present in 9% and harmful use of any substance present in almost half of those studied. Pre-morbid drug use in people with psychosis has been shown to predict longer-term disability [9].

### Treatment factors

The prescription of clozapine, either at the point of admission to rehabilitation or ever in the past, was not associated with discharge. This is an important finding as it does not support the idea that improvement in rehabilitation is largely due to clozapine being prescribed. We found that both antipsychotic polypharmacy and the use of high dose antipsychotics in the past were significantly associated with non-discharge. It is likely that these are related to poorer outcome because they reflect a higher level of treatment resistance in the non-discharged group. Treatment resistance is defined by NICE as the "presence of poor psychosocial and community functioning that persists despite trials of medication that have been adequate in terms of dose, duration and adherence" [2]. An association between the prescription of antipsychotic medication and suicide in people with schizophrenia has been reported and it is likely that this also reflects differences in illness severity [20]. Antipsychotic polypharmacy and high dose prescribing could also be associated with poorer outcome due to an increased likelihood of side effects resulting in poorer functioning. However, the prescription of antipsychotic medication does not guarantee adherence and it may be that the association with poorer outcome reflects poorer compliance and engagement with treatment services in the non-discharged group.

### Social factors

The social factors we examine were not significantly associated with outcome. This is in keeping with other studies, which have shown that social and demographic factors contribute little to predicting outcome in people with psychosis [7,8].

### Illness factors

We noted that admission to rehabilitation within the first ten years of onset of psychosis was more common in the discharged group, although this did not reach statistical significance. This raises the question of whether earlier engagement in rehabilitation might improve outcomes, perhaps for the 10% of people who fail to achieve remission after their first episode of psychosis [7]. This remains an interesting area for future research.

### Changing in-patient characteristics over time

The non-discharged group were admitted later in time and had significantly higher rates of self harm or suicide attempts and higher rates of aggression and violence. They were significantly more likely to have previously been cared for in forensic psychiatric services. The characteristics of the people admitted to the Rehabilitation Service over time have changed, with a trend towards increased levels of challenging behaviour and more highly treatment resistant illness. This is likely to be because of the drive in Scotland to managing this challenging population in conditions of lower security. Scottish government policy recognised that people were admitted to the High Secure State Hospital for longer than was necessary due to a lack of effective local arrangements for mentally disordered offenders [22]. As well as this, the Mental Health (Care and Treatment) (Scotland) Act 2003 allowed people to appeal against being detained in conditions that they felt were excessively secure: the "least restrictive alternative" [23]. Both of these have led to a cut in high secure provision in Scotland and may have led to a greater proportion of people with significant forensic histories entering the rehabilitation system.

### Limitations

We did not measure and correct for symptom severity. It may be that some of the associations of non-discharge are a result of more severe illness. However, we minimised this effect by matching the cases and controls by ward environment and demographically the two groups were similar. We didn't look at the process of rehabilitation and the interventions - for example skills training, family interventions and psychological therapies - that took place. These interventions would be expected to have an impact on outcome. The use of discharge as the primary outcome measure does have limitations as well as the benefits described above. It may not accurately reflect the level of disability [10] and does not include patients' own perceptions of their functioning.

## Conclusions

In a sample of rehabilitation service in-patients, we found that self harm, suicide attempts and previous care in forensic psychiatric services were all significantly associated with not having achieved discharge during the six year period we studied. Non-discharge was also associated with previous treatment with high dose antipsychotics and antipsychotic polypharmacy. This is likely to reflect higher levels of treatment resistance and possibly poorer engagement in the non-discharged group. There was a change over time in the characteristics of the in-patient rehabilitation population and this has important implications for service design. There is a growing need for secure, longer-term in-patient rehabilitation with high staff to patient ratios and access to a wide variety of therapeutic interventions, particularly for people transferred from forensic services. Secure longer-term rehabilitation could be provided as part of either forensic or rehabilitation services. Different areas will have to meet the challenge of this growing need in different ways depending on the design of their local services.

## Competing interests

The authors declare that they have no competing interests.

## Authors' contributions

JB collected the data, performed the statistical analysis and drafted the manuscript. All authors conceived of the study, participated in the design of the study and read and approved the final manuscript.

## Pre-publication history

The pre-publication history for this paper can be accessed here:

http://www.biomedcentral.com/1471-244X/11/149/prepub
